# Effects of serial passaging of field isolates of Bangladeshi PPR virus in Vero cells on the fusion protein

**DOI:** 10.1186/s13620-025-00298-z

**Published:** 2025-07-01

**Authors:** Md. Saiful Islam Siddiqui, Anja Globig, Md. Mushfiqur Rahman, Md. Rafiqul Islam, Emdadul Haque Chowdhury

**Affiliations:** 1 Department of Anatomy & Histology, Faculty of Veterinary, Animal & Biomedical Sciences, Sylhet Agricultural University, Sylhet, 3100 Bangladesh; 2 Friedrich Loffler Institut, Federal Research Institute for Animal Health, Südufer 10, Greifswald, Insel Riems D-17493 Germany; 3 Office of the Director General, Public Private Partnership, Prime Minister Office, Government of Peoples Republic of Bangladesh, Dhaka, Bangladesh; 4 Department of Pathology, Faculty of Veterinary Science, Bangladesh Agricultural University, Mymensingh, 2202 Bangladesh

**Keywords:** Continual passaging, Virus, Cell culture, Fusion protein, Nucleotide sequence

## Abstract

**Objectives:**

Fusion (F) protein is crucial for facilitating viral entry into host cells and contributes to the virulence of Morbilliviruses. Serial passaging of the *Peste Des Petits Ruminants* virus (PPRV) in nonnative hosts can lead to mutations that potentially reduce pathogenicity. Hence, this study aimed to investigate the effects of serial passaging of a Bangladeshi strain of PPR virus in Vero cells on the Fusion protein and pathogenicity

**Materials and methods:**

PPR viruses were initially isolated from natural PPR outbreaks, confirmed through reverse transcriptase polymerase chain reaction (RT‒PCR), passaged to the 9th passage in Vero cells, sequenced, and preserved in a previous study. The 9th passage virus from the repository was utilized as the viral inoculant for further passaging in Vero cells, and the 60th passage was completed. The presence of PPR viral RNA was confirmed in tissue culture fluid (TCF) by RT‒PCR at different passage numbers. TCF at the 60th passage was sequenced and used for immunogenicity studies via live animal experiments, and subsequent immunity was measured via cELISA.

**Results:**

Comparative analysis of the sequences from the 9th and 60th passages, along with other sequences, revealed substitutions of 14 nucleotides (nts) and 4 amino acids (aa) within the leucine zipper structure of the fusion protein. Notably, live animal experiments demonstrated the occurrence of protective immunity.

**Conclusion:**

This study suggests that amino acid substitution and genetic divergence may positively affect viral virulence, highlighting their importance in the development of a potent vaccine.

## Introduction

The fusion protein of the PPR virus (546 amino acids, MW of 59.137 kDa, conserved and GC-rich protein), along with hemagglutinin (H), remains in the envelope and pokes out as spikes. In addition to hemagglutinin, fusion proteins promote viral and mammalian cell surface fusion, thus mediating viral entry and ultimately facilitating entry of the viral genome into the host cell [3, 4, 15, 24, 33]. F_0_, an inactive precursor, is an essential antigenic determinant of morbillivirus virulence and is required for viral infectivity [16, 20, 28, 30, 31, 34]. F_1_ and F_2_ are the two active fractions of F_0._ They are tightly bound by disulfide bonds.

There were four well defined conserved motifs of F_1._ These are (i) the N-terminus fusion peptide (FP) and (ii). Transmembrane (TM) domain (iii). Heptade repeats 1; and (iv). Heptade repeat 2 [12]. They form an asix-helix bundle as a heterodimer between HR2 and HR1, which covers the inner core of the HR1 trimer. F_0_ protein sequence analysis revealed high conservation among the members of the genus Morbillivirus, except for two variable hydrophobic (N- and C-terminal) domains, which serve as conserved regions among all morbilliviruses. The cleavage site of Morbillivirus includes RRX1X2R (where X1 = any amino acid and X2 = either arginine or lysine), and PPR viruses bear RRTRR at positions 104–108 [5, 34]. Viral entry into the host cell depends upon the attachment of the FP domain and dimerization of the HR domains [7–9, 13, 22, 25]. The leucine zipper motif (position 459–480), another conserved fraction of the fusion protein of morbillivirus, also regulates the fusion function of virus particles to the host cell membrane [23].

Sequential passaging *of Peste des petits ruminant* (PPR) viruses in nontraditional hosts such as Vero cells has been suggested as a potential cause for point mutations, leading to reduced pathogenicity. This decrease in pathogenicity is crucial for the development of effective vaccine strategies. Hence, the objective of this study was to examine the impact of serial passaging of the local Bangladeshi strain of PPR virus in Vero cells on the F gene and protein sequences, as well as the virulence of the virus.

## Materials and methods

### Ethics approval

All methods were carried out in accordance with the relevant guidelines and regulations of the “Animal Experimentation and Ethics Committee”, Bangladesh Agricultural University, Bangladesh. All methods were reported in accordance with the ARRIVE guidelines (https://arriveguidelines.org).

### Virus

A Bangladeshi field isolate (BD_PPR_08) was obtained from the virus depository and was serially passaged in Vero cells. TCF at the 60^th^ passage level was used for sequencing.

### Tissue cultures

Imported Vero cells (African Green Monkey Kidney Cells) from the 82^nd^ subculture from Cell Line Services (CLS), Germany (continuous cell line; order no. 605372, vol.− 1.5 ml; glass ampoule) were used for this study.

#### Cell culture media and reagents

In this study, the cell culture medium used was Dulbecco's modified Eagle’s medium (DMEM) supplemented with fetal bovine serum (FBS) and 0.25% trypsin with EDTA. DMEM and FBS were obtained from Gibco-Invitrogen (USA).

#### Procedures for Vero cell culture and subculture

A protocol described by Ammerman et. al*.* [1] was used for culture and subculture of Vero cells.

### Confirmation of PPR viral RNA in TCFs

The presence of viral RNA in the tissue culture fluid (TCF) was confirmed via both conventional RT‒PCR and real-time RT‒PCR at regular intervals, specifically every five passages. These molecular techniques were employed to detect and quantify viral RNA in the TCF, providing valuable insights into the persistence and abundance of the viral genetic material during the serial passaging process.

#### Primers used for RT‒PCR in Bangladesh

The primers used in Bangladesh for amplification of the F gene via RT‒PCR are shown in Table 1.

**Table 1:** Primers were used for RT-PCR in Bangladesh

Gene	Primer	Sequence	Position	Size	References
F gene	PPRV F1b	5´-AGTACAAAAGATTGCTGATCACAGT- 3´	760–784	448 bp	Forsyth and Barrett [10]
	PPRV F2 d	5´-GGGTCTCGAAGGCTAGGCCCGAATA- 3´	1207–1183		

#### Primers used for RT‒PCR in Germany

For amplification of the PPR viral RNA of the N and F genes, the following primers were used in FLI, Germany (Tables 2 & 3).

**Table 2:** Primer (forward) used in FLI, Germany

Mix	Primer name	Primer FOR	Product length	Reference
4	pan-PPRV- 5588 F (FLI)	GCC AGA TTC ACT GGG GCA ATC	1000	FLI
5	pan-PPRV- 6428 F (FLI)	CMT ACA AYA TTG GGG CAC AGG A	1075	FLI

**Table 3:** Primer (reverse) used in FLI, Germany

Mix	Primer name	Primer REV	Product length	Reference
4	pan-PPRV- 6588R	CTG RAG CAA TGG GCT CAT TGG	1000	FLI
5	pan-PPRV- 7503R	CCC TGC AAT GGC CAG CAG	1075	FLI

### Molecular characterization/sequencing

#### Methods

At the 60^th^ passage, the tissue culture fluid (TCF) was sent to the Friedrich Loeffler Institute (FLI) in Germany for sequencing, after which the cell culture-adapted partial sequences of the F gene (1723 nucleotides; BH126/15 - 1_cellculture_60 thpassage_F) and the N gene (931 nucleotides) were obtained. For comparison with cell culture-adapted sequences, the original sequence of the F gene at the 9^th^ passage level from the BD_PPR_08 isolate, obtained in a previous study by Mushfique in 2013 (GenBank accession No. HQ898003), was downloaded from GenBank, along with other related sequences. These sequences were compared with the cell culture-adapted sequences obtained in the present study.

### Homology study and phylogenetic analysis

Lasergene DNA star” (Modules-Edit Seq and MegAlign) and MEGA (Version 6.0) software were used for sequence editing, alignment, nucleotide substitution analysis, homology studies, and phylogenetic analysis.

### Live animal experimentation for immunogenicity studies

Live goats were vaccinated via serially passaged Vero cells adapted to PPRV-TCF at the 60^th^ passage before and after freeze-drying or lyophilization.

#### Materials

##### Experimental goats

Randomly selected and locally collected goats free of PPR antibodies (four goats for animal experiment I and seven goats for animal experiment II) were used in this study.

##### Tissue culture virus (TCV)/fluid

The 60^th^ passage PPRV-TCF for animal experiment I and the vaccine (lyophilized 60^th^ passage PPRV-TCF) for animal experiment II were used for animal inoculation.

#### Methods

##### Infection/inoculation of goats

**Experimental design: animal experiment-I**

Four (04) unvaccinated healthy goats were vaccinated with 60^th^ passage TCF @ 1 ml S/C (6.5 Log_10_TCID_50_/ml), and sera were collected 21 days postvaccination and analysed via cELISA.

**Experimental design: animal experiment-II**

**Lyophilization of 60** ^**th**^ **passage PPRV-TCF**

For lyophilization, the 60^th^ passage PPRV-TCF (Isolate BD_PPR_08) TCF was mixed with stabilizer medium at a ratio of 1:1. The glass ampoules were filled with viral mixture and loosely capped. Finally, the ampoules were placed in a freeze-drying machine for 72 h.

**Animal vaccination**

Five healthy goats were vaccinated with a lyophilized vaccine (freeze-dried 60 th passage TCF) at 1 ml S/C, and sera were collected 21 days postvaccination and analysed via cELISA.

**Challenge infection**

In this study, a total of five vaccinated goats that received freeze-dried or lyophilized vaccines were selected. Additionally, two control goats that did not receive any vaccine were included. All seven goats were inoculated with wild PPRV (Peste des petits ruminants virus) via a 20% weight/volume lymph node tissue homogenate. The goats were closely observed and monitored for any clinical signs and symptoms that may arise following inoculation. The purpose of these observations was to assess the effectiveness of the vaccination in preventing or reducing the occurrence of clinical manifestations associated with PPRV infection.

##### Blood collection, preparation of sera and analysis of sera by competitive ELISA

Whole blood samples were collected from all experimental goats via jugular vein puncture without anticoagulant addition in two steps: before vaccination and 21 days postvaccination. Sera were separated by centrifugation at 3000 rpm for 10 min, transferred to small vials, stored at − 20 °C and then used for competitive ELISA following the protocol of Choi et al. [6].

## Results

### Continual passaging of field isolates of PPR virus in Vero cells

For the adaptation of PPRV in Vero cells, the BD_PPR_08 isolate was serially passaged up to the 60^th^ passage. The cellular changes varied greatly between the 9^th^ and 60^th^ passages, such as increased numbers of intracytoplasmic and intranuclear inclusion bodies, the development of larger syncytia, and the complete degradation of infected cell monolayers, which are suggestive of adaptation and substitutions of the viral fusion protein of the PPR virus in Vero cells.

### Confirmation of viral RNA in tissue culture medium

**Fig. 1:**
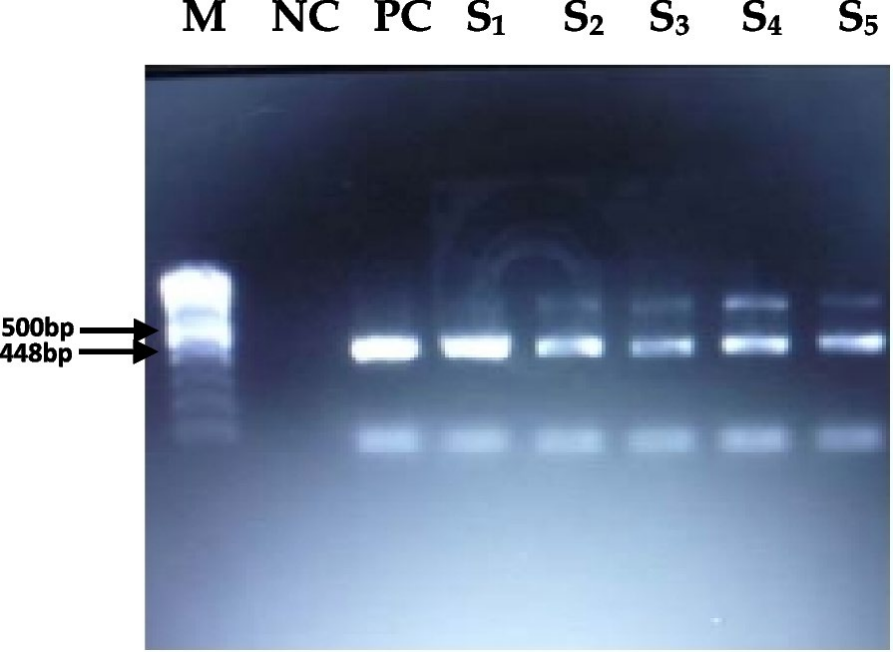
Illustrating the presence of PPR viral RNA of F gene in tissue culture fluid at different passage level. Amplification of 448 bp fragment of F gene by RT-PCR. S1 = 10^th^ passage, S2 = 20^th^ passage, S3 = 30^th^ passage, S4 = 40^th^ passage, S5 = 50^th^ passage PPRV infected TCF. Lane M: 100 bp DNA size marker, lane NC: negative control, Lane PC: positive control

**Fig. 2:**
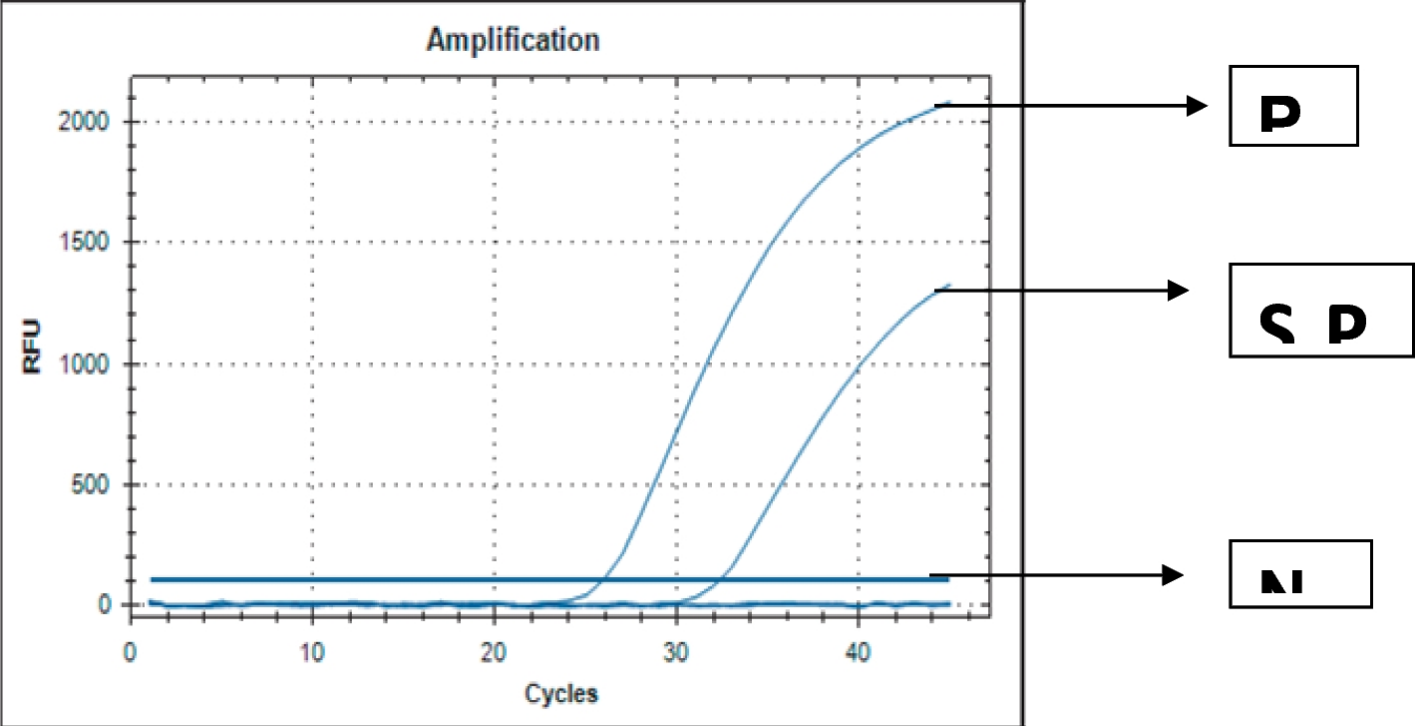
Confirmation of presence of PPR viral RNA of F gene at 60^th^ Passage level by Real Time RT-PCR amplification of PPR viral RNA taken from TCF at 60^th^ Passaged level. Here, PC = Positive control, NC = Threshold line, S = Sample (BD_PPR_08 isolate)

**Fig. 3:**
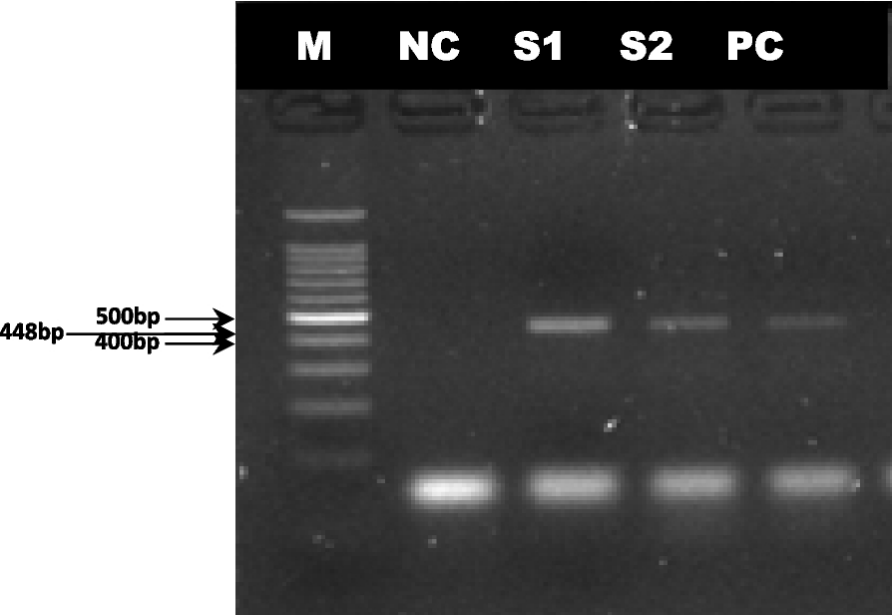
Confirmation of presence of PPR viral RNA of F gene at 60^th^ Passage level. Amplification of 448 bp fragment of F gene by RT-PCR. RNA was extracted from TCF at 60^th^ passage level. Here, Lane M = 100 bp DNA size marker, lane NC = negative control, Lane PC = positive control, Lane S1 = 55^th^ Passage and S2 = 60^th^ passage TCF, TCF = Tissue culture fluid

Viral RNA was detected in TCFs at different passage numbers, such as the 10^th^, 20^th^, 30^th^, 40^th^, and 50^th^ passages (Fig. 1) and the 55^th^ and 60^th^ passages (Fig. 3). However, at the 60^th^ passage, viral RNA was detected by both real-time and conventional RT‒PCR (Figs. 2 & 3). Real-time RT‒PCR was performed for confirmation and to avoid controversy regarding the nonspecific amplification of viral RNA by conventional RT‒PCR.

### Molecular characterization of the studied PPR virus isolate

**Fig. 4:**
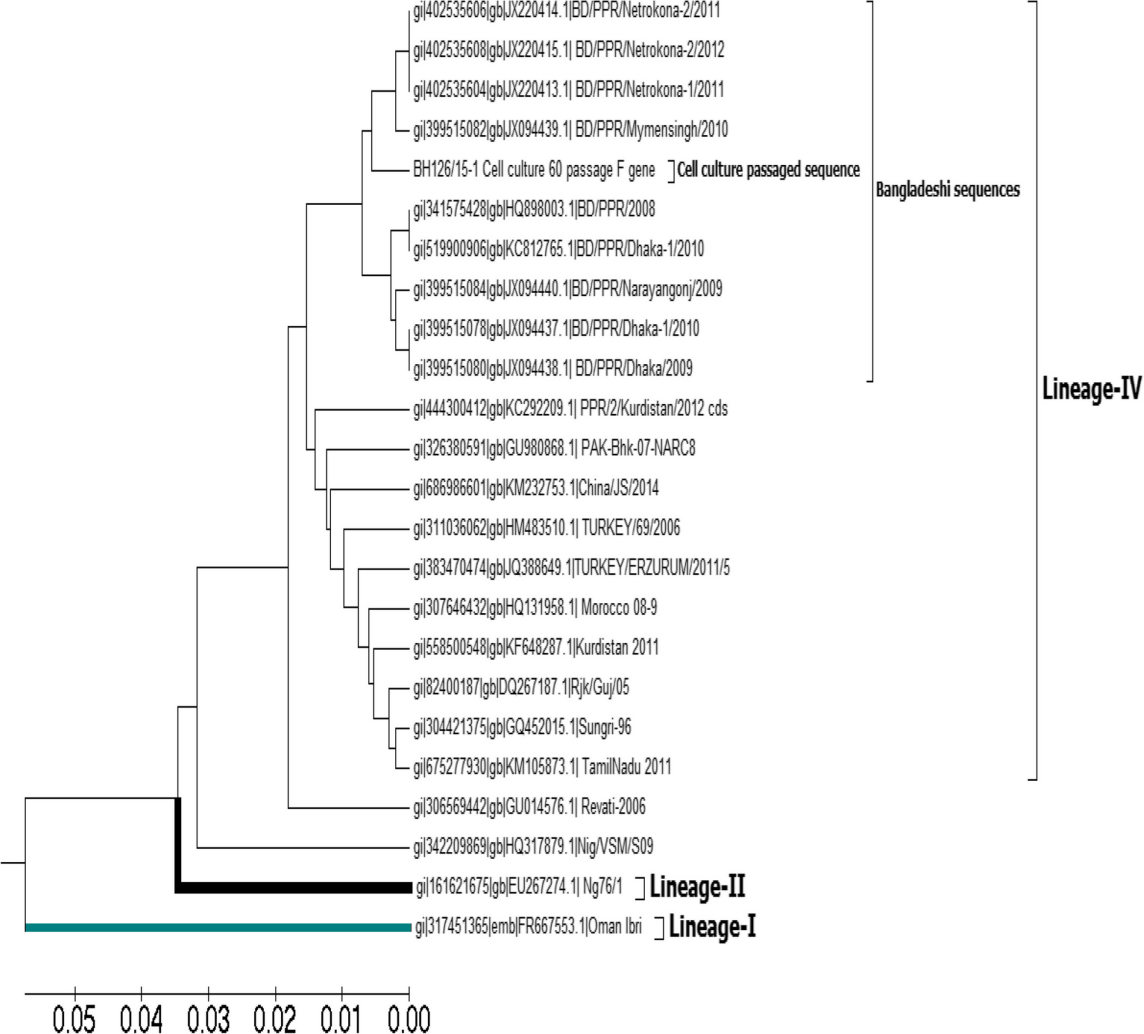
Evolutionary relationships among isolates of different PPR virus based on partial F gene sequences showing that, cell culture passaged sequence at both 9^th^ and 60^th^ passage level form same cluster with other Bangladeshi sequences under the same lineage (lineage-IV)

**Fig. 5:**
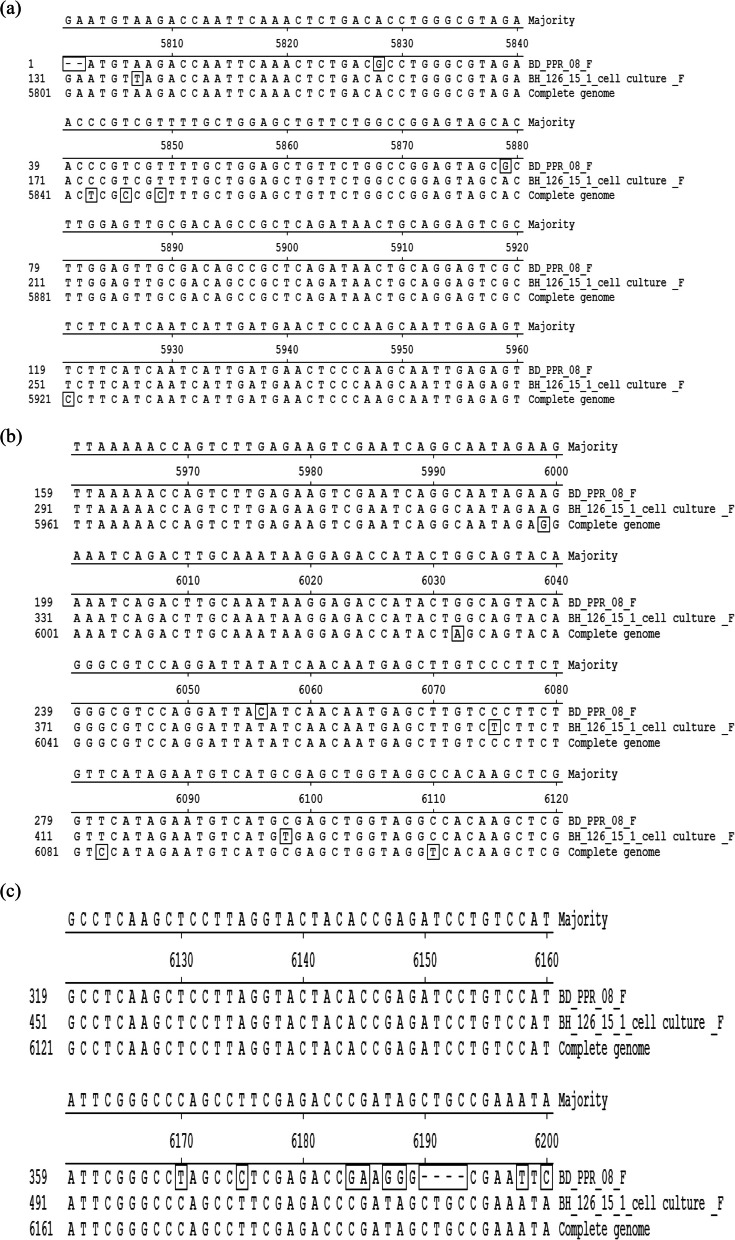
**a**, **b**, **c** The positions of substituted nucleotides of sequence at 60^th^ passage level. Here, BD_PPR_08 = partial nucleotide sequences of F gene at 9^th^ passage level (BD_PPR_08 (Genebank accession No. HQ898003), BH _126_15_1 cell culture_F = Nucleotide sequence of serially passaged BD_PPR_08 isolate at 60^th^ passage level. Alignment of deduced amino acid sequence of F protein. Residues that did not match to the consensus are indicated by box. Alignment of nucleotide sequence of F gene. Residues that did not match to the consensus are indicated by box

Later, the virus from the TCF of the 60^th^ passage was sequenced at FLI, Germany. The partial F gene of PPRV from the same isolate at the 9^th^ passage level was sequenced previously by the same research group [19] (GenBank accession no. HQ 898003). The two sequenced datasets (at the 9^th^ and 60^th^ passage levels) of the partial F gene were aligned and analysed to identify potential point mutations that occurred due to serial passaging (Figs. 4 and 5a, b, c).

### Phylogeny

Partial nucleotide sequences of the F gene at the 9^th^ passage level (BD_PPR_08 (GenBank accession No. HQ898003; nt = 394 bp) and the cell culture-adapted sequence at the 60^th^ passage level (BH126/15–1 cell culture; nt = 1723 bp) and wild-type strains of PPRV (complete genome sequence; nt = 1641 bp) were aligned, and a phylogenetic tree was constructed via the UPGMA method (Fig. 4). BH126/15 - 1_ cells remained in the same subcluster together with other Bangladeshi field strains, such as BD/PPR/Netrokona- 1/2011, BD/PPR/Netrokona- 2/2011, BD/PPR/Netrokona- 2/2012, and BD/PPR/Mymensingh/2010 (GenBank accession No. JX220413.1 JX220414.1, JX220415.1 and JX094439.1, respectively), and the 9^th^ passaged same/original isolate BD_PPR_08 (Gen Bank accession No. HQ898003) (Fig. 4) formed a separate subcluster indicating genetic divergence.

### Nucleotide substitution analysis of the F gene

Fourteen nucleotides were found to be substituted between positions 5807 and 6200 (Table 4). The alignment of the nucleotide sequence of the F gene is shown in detail in Fig. 5a, b, c.

**Table 4:** List of nucleotides substituted with their position

Positions	Nucleotides	Substituted by
5807	A	T
5828	G	A
5879	G	A
6056	C	T
6075	C	T
6098	C	T
6170	T	C
6175	C	T
6184	G	C
6185	A	G
6187	G	T
6188	G	A
6198	T	A
6200	C	A

### Analysis of amino acid substitutions

**Fig. 6:**
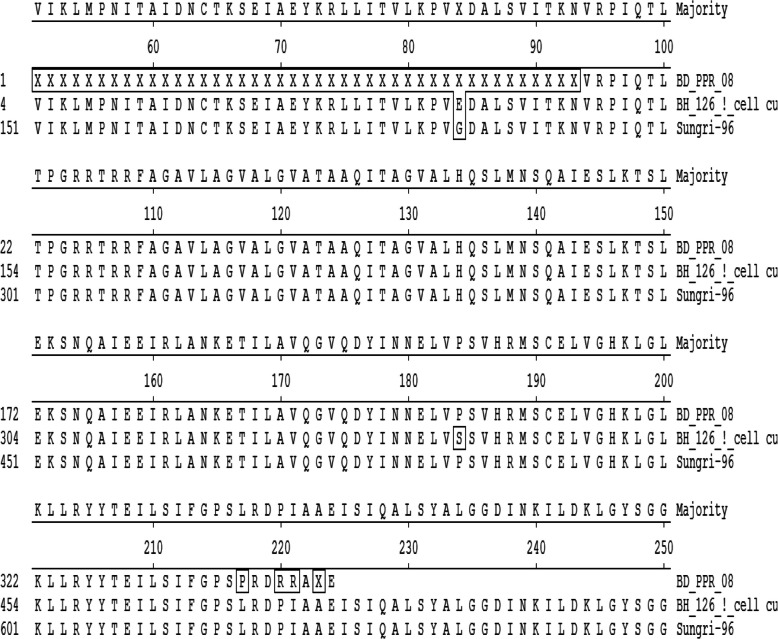
The positions of substituted amino acid of sequence at 60^th^ passage level. Here, BD_PPR_08 = protein sequence of F protein at 9^th^ passage level (BD_PPR_08), BH_126_15_1 cell culture_F = protein sequence of F protein of serially passaged BD_PPR_08 isolate at 60^th^ passage level. Alignment of deduced amino acid sequence of F protein. Residues that did not match to the consensus are indicated by box

Four (4) amino acid substitutions were found in the F_1_ subunit at positions 338, 471, 474 and 475, three of which were found at the cleavage site, particularly in the “leucine zipper” structure of the F_1_ subunit (at positions 459–480 aa) (Table 5). An alignment of the F protein sequence is shown in detail in Fig. 6.

**Table 5:** List of substituted amino acids with their positions

Positions	Amino acids	Substituted by
338	P	S
471	P	L
474	R	P
475	R	I

### Similarities and divergence analysis

**Fig. 7:**
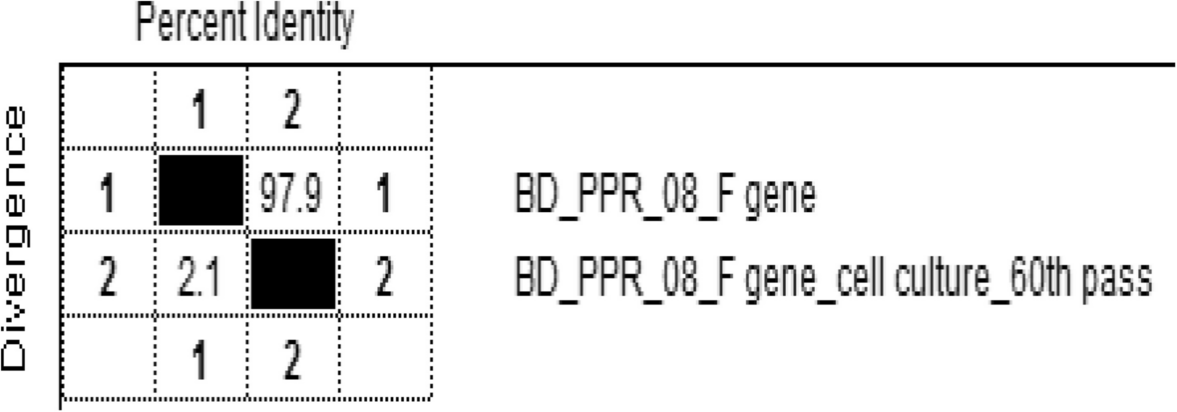
Divergence and similarities between cell culture passaged F gene sequences at 9^th^ and 60^th^ passaged level of isolate BD_PPR_08

A total of 97.9% similarity and 2.1% divergence were found between the study sequences (Fig. 7).

### Study of the immunogenicity of TCF by live animal experimentation

To study the protective efficacy and immune response of vaccine candidates, live animal experiments were conducted in goats, which are free of PPR viral antibodies. In the animal experiment-I, tissue culture fluid (PPRV-TCF) at the 60^th^ passage level was used, and in the animal experiment-II, a lyophilized vaccine (lyophilized PPRV-TCF at the 60^th^ passage level) was used.

#### Animal experiment-I

Four (4) healthy PPRV antibody-free goats were vaccinated subcutaneously with 1.0 ml/goat of the 60^th^ passaged PPRV-TCF (6.5 log_10_TCID_50_/ml). After 21 days of vaccination, serum samples were collected, and PPRV-specific antibody titres were measured via competitive ELISA (c-ELISA). The OD value, competition percentage (CP), and seroconversion percentage are shown in Table 6. Serially passaged and Vero cell-adapted PPRV-TCF resulted in 100% seroconversion in inoculated goats (Table 6).

**Table 6:** Table shows 100% sero-conversion against attenuated PPRV in experimental goats (experiment-I)

Goat no	OD Value of Sample	CP Value	Average	Remarks (Reference value: ≤ 35 = + Ve)	Sero-conversion (%)
1	0.29	16.17	17.44	Positive (P)	100%
2	0.24	13.32		P	
3	0.35	18.91		P	
4	0.39	21.34		p	

#### Animal experiment II

Five (5) healthy PPRV antibody-free goats were vaccinated with the test lyophilized vaccine after being diluted with conventional diluents (LRI diluents) at 1.0 ml/goat subcutaneously, and sera were collected 21 days after vaccination. PPRV-specific antibody titres were measured via c-ELISA. The OD values, competition percentages (CPs), and seroconversion percentages are listed in Table 7. Serially passaged and Vero cell-adapted lyophilized PPR virus vaccines provided 100% seroconversion (Table 7).

**Table 7:** Table shows 100% sero-conversion against lyophilized attenuated PPRV in experimental goats (experiment-II)

Goat no	OD Value of Sample	CP Value	Average	Remarks (Reference value: ≤ 35 = + Ve)	Seroconversion (%)
1	0.22	12.94	14.03	Positive (P)	100%
2	0.29	17.58		P	
3	0.23	13.64		P	
4	0.20	12.00		P	
5	0.23	14.00		P	

##### Challenge experiment

**Fig. 8:**
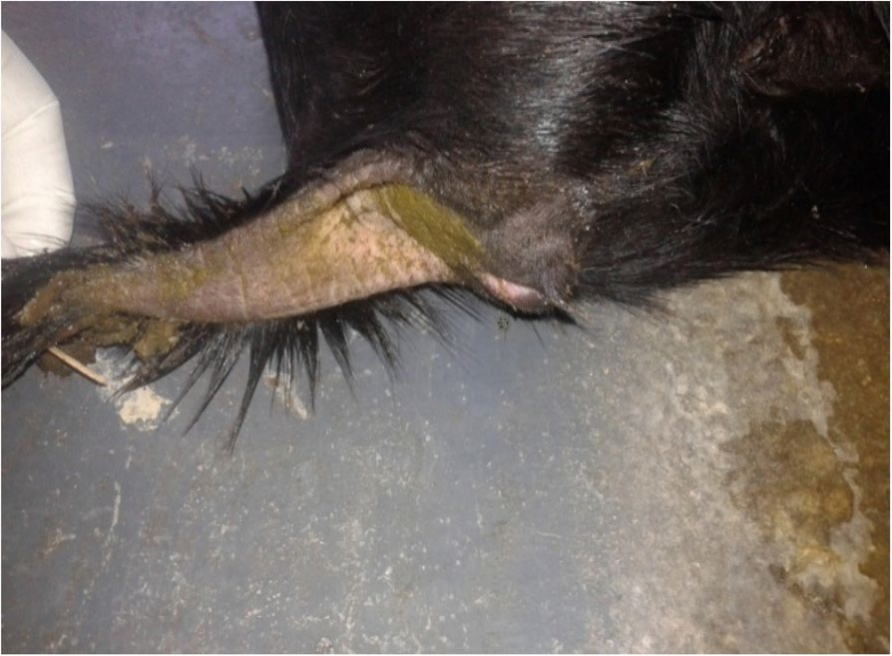
Unvaccinated goats showed clinical sign (diarrhoea) suggestive to PPR in challenge infection (live animal experimentation)

**Fig. 9:**
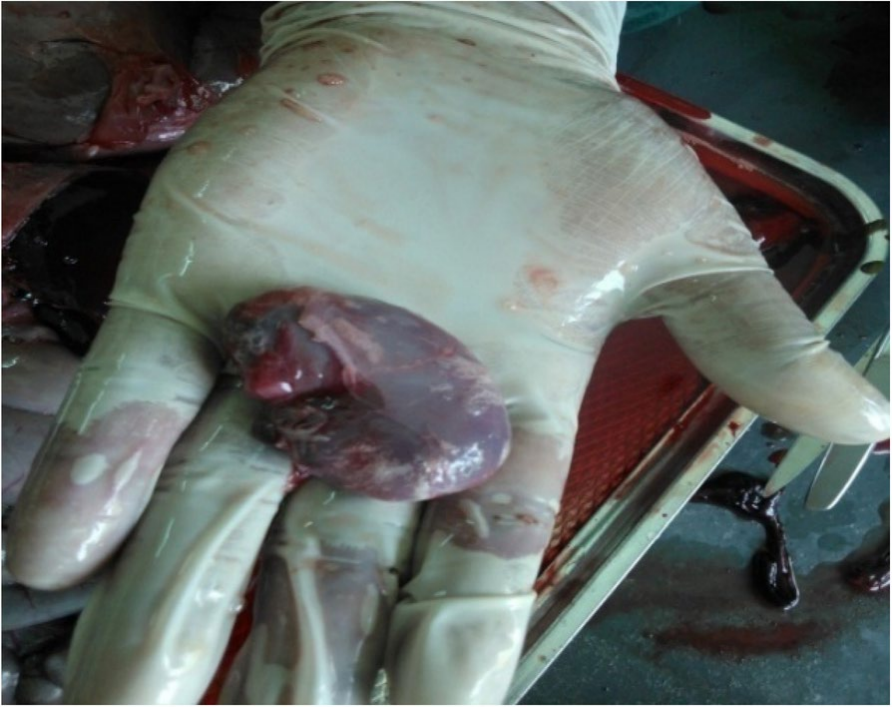
Unvaccinated goats showed pathological changes (swollen hemorrhagic lymph node) suggestive to PPR in challenge infection (live animal experimentation)

**Fig. 10:**
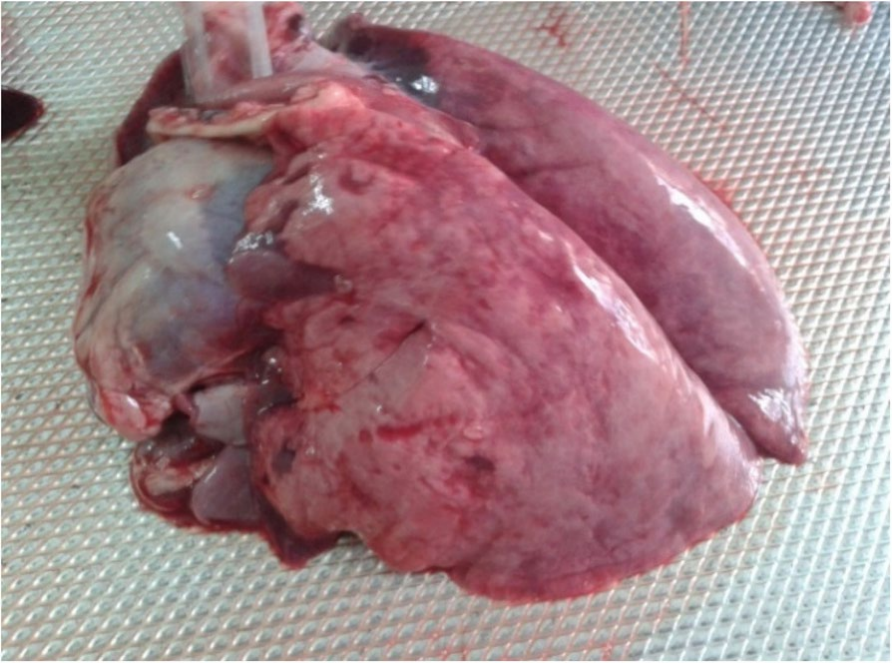
Unvaccinated goats showed pathological changes (congested pneumonic lungs) suggestive to PPR in challenge infection (live animal experimentation)

**Fig. 11:**
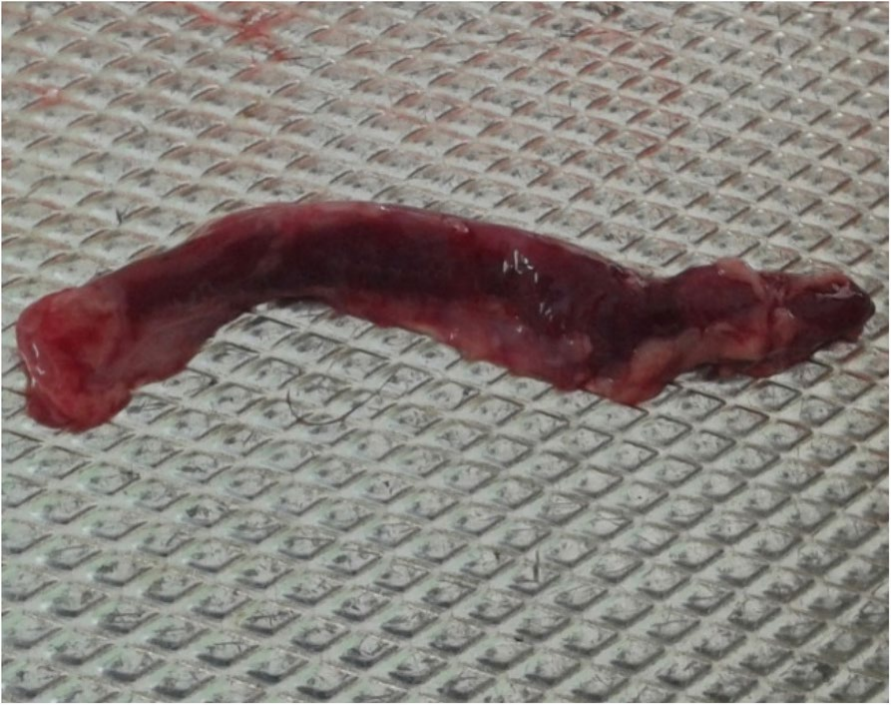
Unvaccinated goats showed pathological changes (haemorrhagic enteritis in intestinal mucosa) suggestive to PPR in challenge infection (live animal experimentation)

Seven PPRV antibody-free goats were selected for the challenge experiments. Five goats were vaccinated with the lyophilized vaccine as described above, and another two goats were used as the unvaccinated control. After 10 days, both the vaccinated and control goats were infected with the wild-type PPR virus and observed for clinical signs and mortality, if any. The vaccinated goats did not show any clinical signs suggestive of PPR, whereas the two unvaccinated goats showed clinical PPR and died. The clinical and pathological changes observed in dead goats included diarrhea (Fig. 8), swollen and hemorrhagic lymph nodes (Fig. 9), congested lungs (Fig. 10), and hemorrhagic intestinal mucosa (Fig. 11).

## Discussion

A phylogenetic tree was constructed via the UPGMA method [11], which revealed that the sequence at the 60^th^ passage level (BH126/15 - 1_cell culture) remained in the same cluster as other Bangladeshi sequences under Lineage IV (BD/PPR/Netrokona- 1/2011 (GenBank accession No JX220413.1), BD/PPR/Netrokona- 2/2011 (GenBank accession No JX220414.1), BD/PPR/Netrokona- 2/2012 (GenBank accession No JX220415.1), and BD/PPR/Mymensingh/2010 (GenBank accession No JX094439.1). This might be due to lineage similarities among isolates and their geographical distribution, as reported by Moudgil et al. [17]. To overcome adverse situations, unusual host genetic mutations can occur as a consequence of serial passaging. This may also occur in a hostile environment, where the virus adapts via mutation. This phenomenon occurred in this study and is in agreement with the findings of Zhu et al. [36] and Munduganor et al. [18]. Homology analysis revealed 97.9% similarity and 2.1% divergence for the F gene as a result of the substitution of a few nucleotides (14 nucleotides) and amino acids (04 aa), which supports the findings of Raoof [26], Xia et al. [35] and Manzoor et al. [14], with little variation. In another study conducted by Peletto et al. [21], dolphin morbilli virus (DMV) was cultured in Vero DogSLAM tag cells, the complete sequence was analysed, and more amino acid (47 amino acid) substitutions were found, this large variation might be due to the analysis of the full-length sequence. A limitation of this study is that the sequences were not equal in length. Four (04) aa were substituted in this study from the F_1_ subunit, including three (03) from the leucine zipper structure. It has been established that any impairment of the leucine zipper structure and F_2_ glycosylation site could abolish the pathogenicity of the PPR virus. Amino acid substitution is an indication of adaptation and attenuation, which may be due to point mutations. This point mutation may be due to serial passaging of the virus in an unconventional host, which supports the findings of Balamurugan et al*.* [2] and Dhar et al*. *[9]. Live animal experiments revealed protective immunity, which is in accordance with the studies of Sreenivasa et al. [32], Choi et al. [6], Saravanan et al. [27], and Sen et al. [29]. In conclusion, serial passaging of field isolates of the PPR virus in Vero cells results in amino acid substitution and genetic divergence, which might decrease viral virulence, highlighting its importance in the development of effective vaccine candidates.

## Conclusion

In conclusion, the results of this study suggest that the substitution of amino acids and genetic divergence resulting from serial passaging can potentially reduce the virulence of the PPR virus, which reflects the production of protective immunity. These findings hold importance in the development of a potent, effective vaccine candidate against the virus via the use of local isolates.

## Data Availability

Sequence data for the fusion protein of the PPR virus were generated in this study. All the data generated or analysed during this study are included in this published article [and its supplementary information files; namedraw data files] "in the submission system.

## Abbreviations

aa: Amino acid
EDTA: Ethylenediamine Tetra amine Acetic acid
F: Fusion
F_1_ and F_2_: F_1_ and F_2_ subunits
FBS: Fetal bovine serum
FLI: Friedrich Loeffler Institute
kDa: Kilo Dalton
nt_s_: Nucleotides
TCF: Tissue culture fluid
